# Update of the treatment of nosocomial pneumonia in the ICU

**DOI:** 10.1186/s13054-020-03091-2

**Published:** 2020-06-29

**Authors:** Rafael Zaragoza, Pablo Vidal-Cortés, Gerardo Aguilar, Marcio Borges, Emili Diaz, Ricard Ferrer, Emilio Maseda, Mercedes Nieto, Francisco Xavier Nuvials, Paula Ramirez, Alejandro Rodriguez, Cruz Soriano, Javier Veganzones, Ignacio Martín-Loeches

**Affiliations:** 1 Critical Care Department, Hospital Universitario Dr. Peset, Valencia, Spain; 2 Fundación Micellium, Valencia, Spain; 3 ICU, Hospital Universitario Ourense, Ourense, Spain; 4 SICU, Hospital Clínico Universitario Valencia, Valencia, Spain; 5 ICU, Hospital Universitario Son Llázter, Palma de Mallorca, Spain; 6 Department of Medicine, Universitat Autònoma de Barcelona (UAB), Barcelona, Spain; 7 Critical Care Department, Corporació Sanitària Parc Taulí, Sabadell, Barcelona, Spain; 8 CIBERES Ciber de Enfermedades Respiratorias, Madrid, Spain; 9 ICU, Hospital Vall d’Hebrón, Barcelona, Spain; 10 SICU, Hospital Universitario La Paz, Madrid, Spain; 11 ICU, Hospital Clínico Universitario San Carlos, Madrid, Spain; 12 ICU, Hospital Universitari I Politecnic La Fe, Valencia, Spain; 13 ICU, Hospital Universitari Joan XXIII, Tarragona, Spain; 14 ICU, Hospital Universitario Ramón y Cajal, Madrid, Spain; 15 ICU, Trinity Centre for Health Science HRB-Wellcome Trust, St James’s Hospital, Dublin, Ireland

**Keywords:** HAP, VAP, Nosocomial pneumonia, Ceftolozane-tazobactam, Ceftazidime-avibactam, *Pseudomonas aeruginosa*, KPC, PCR

## Abstract

In accordance with the recommendations of, amongst others, the *Surviving Sepsis Campaign* and the recently published European treatment guidelines for hospital-acquired pneumonia (HAP) and ventilator-associated pneumonia (VAP), in the event of a patient with such infections, empirical antibiotic treatment must be appropriate and administered as early as possible. The aim of this manuscript is to update treatment protocols by reviewing recently published studies on the treatment of nosocomial pneumonia in the critically ill patients that require invasive respiratory support and patients with HAP from hospital wards that require invasive mechanical ventilation. An interdisciplinary group of experts, comprising specialists in anaesthesia and resuscitation and in intensive care medicine, updated the epidemiology and antimicrobial resistance and established clinical management priorities based on patients’ risk factors. Implementation of rapid diagnostic microbiological techniques available and the new antibiotics recently added to the therapeutic arsenal has been reviewed and updated. After analysis of the categories outlined, some recommendations were suggested, and an algorithm to update empirical and targeted treatment in critically ill patients has also been designed. These aspects are key to improve VAP outcomes because of the severity of patients and possible acquisition of multidrug-resistant organisms (MDROs).

## Introduction/methodology

In accordance with the recommendations of, amongst others, the *Surviving Sepsis Campaign* [1] or the latest European treatment guidelines for hospital-acquired pneumonia (HAP) and ventilator-associated pneumonia (VAP) [2], in the event of a patient with such infections, empirical antibiotic treatment must be appropriate and administered as early as possible. Complying with these conditions is more important and more complex in patients being admitted to an intensive care unit (ICU), both because of the severity of patient and the potential acquisition of multidrug-resistant organisms (MDROs) which will doubtlessly be related to a higher level of unsuitable empirical treatment and, consequently, higher mortality. As an example, when reviewing the data from the National Surveillance Programme of Intensive Care Unit (ICU)-Acquired Infection in Europe Link for Infection Control through Surveillance (ENVIN-HELICS) [3], the likelihood of receiving an inadequate empirical treatment for a *Pseudomonas aeruginosa* infection, even with combination therapy, is approximately 30%.

The development of new antibiotics and their use should be cautious. In the present manuscript, we propose different algorithms that allow to implement empirical and targeted use for potential MDROs. We must first and foremost capitalize on their greater in vitro activity, lower resistance and suitable efficacy in clinical trials and, secondly, antibiotic diversification and the need for carbapenem-sparing strategies [4, 5]. Antimicrobial optimization programmes, such as the *US antimicrobial stewardship programmes* (ASP), aim to improve the clinical outcomes of patients with nosocomial infections, minimizing adverse effects associated with the use of antimicrobials (including the onset and dissemination of resistance) and guaranteeing the use of cost-effective treatments [6]. In addition, the analysis of its use and results obtained in patients and microbiological resistance result paramount. Avoiding unnecessary treatments and reducing the spectrum and duration of treatment together with the reduction of adverse effects and/or possible interactions will be the ultimate aim [7, 8].

This point of view article summarizes the recently published literature on the management of nosocomial pneumonia in the critically ill patients that require invasive respiratory support, both those arising from hospital wards that ultimately require ICU admission and those associated with mechanical ventilation. Experts were selected on the basis of their contrasted experience in the field of nosocomial infections, including specialists in anaesthesia and in intensive care medicine. An extensive search of the literature was performed by the authors using the MEDLINE/PubMed and Cochrane library databases, from 2009 to October 2019, aimed to retrieve relevant studies on diagnosis and treatment of nosocomial pneumonia in ICU patients especially randomized controlled clinical trials (RCT), systematic reviews, meta-analysis and expert consensus articles. Priorities have been established in regard to the management, agreed by the group and based on risk factors for their development and prognostic factors. Moreover, the most important clinical entities, methods of rapid diagnostics in clinical microbiological available and new antibiotic treatments recently added to the therapeutic options have been reviewed and updated. After the analysis of the priorities outlined, recommendations that can be applied have been included. An algorithm that takes into account the priorities analysed to update empirical and targeted treatment in ICUs has also been designed.

## Epidemiology

The definitions of hospital-acquired pneumonia (HAP) and ventilator-associated pneumonia (VAP) are not homogeneous and may alter the incidences reported [9]. In this document, we will refer to HAP as that which appears as of 48 h from hospital admission, in the ICU or in the hospital ward, whether or not related to mechanical ventilation (MV). We will use the term HAP to talk of that HAP unrelated to MV or intubation, as opposed to VAP, which is what appears after 48 h of MV. When a patient presents symptoms of infection of the lower respiratory tract after more than 48 h under MV and does not present opacities on chest X-ray, the patient is diagnosed with ventilator-associated tracheobronchitis (VAT).

Respiratory infections are the most prevalent nosocomial infection observed in ICUs [10]. In a broad global multicentre study, half the patients presented an infection at the time of the observation, 65% of respiratory origin [11] and HAP and VAP accounted for 22% of all hospital infections in a prevalence study performed in 183 US hospitals [12]. A total of 10 to 40% of patients who underwent MV for more than 48 h will develop a VAP. Marked differences are observed between different countries and kinds of ICU [13]. These variations can be accounted for by diagnostic difficulties, differences in the definition used, the diagnostic methods used and the classification of units because the prevalence of VAP is higher in certain populations (patients with adult respiratory distress syndrome (ARDS) [14], with brain damage [15], or patients with veno-arterial extracorporeal membrane oxygenation (VA-ECMO) [16].

If we analyse the density of incidence, significant differences between European and US ICUs have been reported. The National Healthcare Safety Network (NHSN) (2013) reported that the average rate of VAP in the USA was 1–2.5 cases/1000 days of MV [17], substantially lower than in Europe, 8.9 episodes/1000 days of MV according to the European Centre for Disease Prevention and Control (ECDC) [18]. In Spain, according to the ENVIN-HELICS 2018 report, the incidence was 5.87 episodes/1000 days of MV [3]. Both in the USA and in Europe, the incidence of VAP has gradually reduced [19], probably in relation to preventive measures [20], although a potential bias cannot be ruled out due to not very objective monitoring criteria.

A condition with growing relevance is ventilator-associated tracheobronchitis (VAT). In a prospective and multicentre study, the incidence of VAT and VAP was similar with 10.2 and 8.8 episodes for 1000 days of mechanical ventilation, respectively [21]. Sometimes, it is difficult to differentiate VAT and VAP, and in fact, some authors advocate that the two entities are a continuum and that VAT patients can evolve towards VAP [22]. These authors report a series of reasons in their rationale: higher incidence of VAP in patients with VAT compared to those with VAT, post-mortem findings coexisting in both entities, higher ranges of biomarkers (procalcitonin) or severity scores in VAP compared to VAT and mortality, or a common microbiology [23].

Non-ventilated ICU patients appear to have a lower risk of developing pneumonia, as reported in a recent study, where 40% of cases of pneumonia acquired in the ICU occurred in patients who had not been ventilated previously [24]. Another study, performed in 400 German ICUs, reports a number of VAP of 5.44/1000 days MV, as opposed to 1.58/1000 days of non-invasive mechanical ventilation (NIMV) or 1.15/1000 HAP patients [25]. The global incidence (including intra- and extra-ICU) of HAP ranges from 5 to more than 20 cases/1000 hospital admissions, being more complex to determine, because of the heterogeneity of definitions and the methodology used. The European Centre for Disease Prevention and Control (ECDC), analysing data from 947 hospitals in 30 countries, reports a prevalence of HAP of 1.3% (95% CI, 1.2 to 1.3%) [26]. However, a US study reports a frequency of HAP of 1.6% in hospitalized patients, with a density of incidence of 3.63/1000 patients-day [27]. Moreover, a Spanish multicentre study [28] that analysed 165 episodes of extra-ICU HAP reports an incidence of 3.1 (1.3–5.9) episodes/1000 admissions, variable according to hospital and type of patient.

In the non-ventilated patient’s group, when cultures are available, the aetiology is similar to VAP [24], with a predominance of *P. aeruginosa*, *S. aureus* and *Enterobacteriaceae* spp. [29]. This also depends on the patient’s severity, individual risk factors and local epidemiology.

Table 1 summarizes the studies published from 2010 to 2019 about the microbiology of ICU-acquired pneumonia (including HAP, VAP and VAT).

**Table 1:** Microbiology and main resistance profile of microorganism causing VAP, VAT and HAP in non-ventilated patients treated in ICU (data from studies published from 2010 to 2019)

Reference	Type of infection	Microbiology				
Ferrer et al. [30]	HAP	*S. aureus*, 17.7%	*P. aeruginosa*, 17.7%	*E.coli*, 6.5%	*Enterobacter* spp., 4.3%	*K. pneumoniae*, 3.2%
Esperatti et al. [22]	VAP	*P. aeruginosa*, 24%	*S. aureus*, 23%	*E. coli*, 7%	*Enterobacter* spp., 6%	*H. influenzae*, 4%
Restrepo et al. [31]	VAP	*S. aureus*, 38.7%	*H. influenzae*, 23.4%	*P. aeruginosa*, 14.7%	*k. pneumoniae*, 11.5%	*E. coli*, 11.1%
		MDR, 30%				
Quartin et al. [32]*	VAP	*S. aureus*, 60.3%	*P. aeruginosa*, 9.4%	*Acinetobacter* spp., 7.3%	*Klebsiella* spp., 6.8%	*Enterobacter* spp., 5.1%
Nseir et al. [33]	VAT	*P. aeruginosa*, 34.4%	*S. aureus*, 20.5%	*A. baumanii*, 11.5%	*K. oxytoca*, 10.6%	*Enterobacter* spp., 9.8%
		MDR, 36.8%				
Martín-Loeches et al. [21]	VAT	*P. aeruginosa*, 25%	*S. aureus*, 23%	*Klebsiella* spp., 15%	*E. coli*, 12%	*Enterobacter* spp., 11%
		MDR, 61%				
	VAP	*P. aeruginosa*, 24%	*S. aureus*, 24%	*Klebsiella* spp., 14%	*Enterobacter* spp., 12%	*E. coli*, 11%
		MDR, 61%				
ECDC [18]	VAP	*P. aeruginosa*, 20.8%	*S. aureus*, 17.8%	*Klebsiella* spp., 16.1%	*E. coli*, 13.3%	*Enterobacter* spp., 10.3%
Koulenti et al. [29]	HAP	*Enterobacteriaceae*, 32.9%	*S. aureus*, 24.9%	*P. aeruginosa*, 17.4%	*A. baumanii*, 15.4%	
ENVIN-HELICS [3]	VAP	*P. aeruginosa*, 23.8%	*S. aureus*, 13.5%	*Klebsiella* spp., 10.3%	*E. coli*, 9.1%	*Enterobacter* spp., 8.6%
		PIP/TAZ R, 34.1% Carba R, 37.9% Colistin R, 8.6%	MRSA, 12.7%	PIP/TAZ R, 50% Carba R, 23.5% 3°G cef R, 37%	PIP/TAZ R, 21.7% Carba R, 0% 3°G cef R, 12.5%	
Pulido et al. [34]	VAP	*P. aeruginosa*, 21.1%	*A. baumanii*, 17.9%	*K. pneumoniae*, 15.6%	*S. aureus*, 13.3%	*E. coli*, 7.8%
Huang et al. [35]	VAP	*A. baumanii*, 33.9%	*K. pneumoniae*, 23.6%	*P. aeruginosa*, 19.8%	*S. aureus*, 7.1%	*S. maltophilia*, 3.8%
		Carba R, 76.4%	Carba R, 44%	Carba R, 59.5%	MRSA, 60%	
Cantón-Bulnes et al. [36]	VAT	*P. aeruginosa*, 24.5%	*H. influenzae*, 18.9%	*E. coli*, 9.4%	*S. aureus*, 9.4%	*K. pneumoniae*, 7.5%
Ibn Saied et al. [37]	VAP	*P. aeruginosa*, 33.5%	*Enterobacteriaceae*, 32.3%	*S. aureus*, 19%	*S. pneumoniae*, 4.9%	*S. maltophilia*, 4.7%

*carba* carbapenem, *HAP* hospital-acquired pneumonia, *MDR* multidrug resistant, *VAP* ventilator-associated pneumonia, *VAT* ventilator-associated tracheobronchitis, *PIP/TAZ* piperacillin/tazobactam, *R* resistance, *3°G cef* 3° generation cephalosporin

*Trial designed to compare MRSA pneumonia treatment, special effort to include patients with MRSA pneumonia

## Impact on outcome

According to a case-control study, HAP patients presented a worse clinical course: higher mortality (19% vs 3.9%), more ICU admissions (56.3% vs 22.8%) and longer hospital stay (15.9 days vs 4.4 days). Overall, patients with HAP presented an odds ratio of dying 8.4 times higher than non-HAP patients [38]. It has traditionally been considered that VAP-associated mortality is higher than HAP [39]. When ICU-HAP was compared to VAP [25], the crude mortality was similar, which suggests that it is related more to patient-related factors than prior intubation. Therefore, when analysing data from 10 recent clinical trials in ICU patients, mortality was greater for HAP requiring MV, somewhat lower in VAP and less for non-ventilated HAP [40]. The need for intubation in this population is probably a marker of poor clinical progression of pneumonia. Adjusted mortality rates were similar for VAP and ventilated HAP. In a recent multicentre study that includes more than 14,000 patients and investigates the impact of VAP and HAP in the ICU, both were associated with a higher risk of death at 30 days [HR 1.38 (1.24–1.52) for VAP and 1.82 (1.35–2.45) for HAP] [37].

Overall, the mortality from HAP of 13% with an increase in hospital stay of 4 to 16 days and increased cost of 40,000 dollars per episode has been reported [27]. VAP has also been associated with an increased stay in the ICU and hospital, in addition to the increased time under mechanical ventilation [41]. The crude mortality rates of patients with VAP vary between 24 and 72%, with greater mortality in VAP caused by *Pseudomonas aeruginosa* [42]*.* The more recent data estimate attributable mortality of 13%, higher in patients with intermediate severity and in surgical patients [43]. As for VAT, this has been related in different studies to a longer stay in ICU and more days of MV. However, to date, there are no randomized controlled trials showing a beneficial effect for the treatment in VAT. Moreover, higher mortality in patients presenting this complication has not been observed [21, 36, 44].

## HAP risk factors

Traditionally, three kinds of risk factors for nosocomial pneumonia have been considered: patient-related, infection prevention-related and procedures-related. Patient-related factors are acute or chronic severe disease, coma, malnutrition, prolonged hospital length of stay, hypotension, metabolic acidosis, smoking and comorbidities (especially of the central nervous system but also chronic obstructive pulmonary disease (COPD), *diabetes mellitus*, alcoholism, chronic renal failure and respiratory insufficiency). Amongst risk factors related to infection prevention, those notable are deficient hand hygiene or inappropriate care of respiratory support devices. Finally, amongst factors related to procedures, administration of sedatives, corticosteroids and other immunosuppressants, prolonged surgical procedures (especially at thoracic or abdominal level) and prolonged/inappropriate antibiotic treatment are the most recognized factors [13, 38, 45–47]. More recent studies have observed an increased risk of nosocomial pneumonia in patients who receive gastric acid-modifying drugs during their admission (OR: 1.3 [1.1–1.4]) [48].

Given that there is no artificial airway, we can consider pneumonia in the patient who undergoes NIMV as a subtype of pneumonia in the non-ventilated patient. A prospective study analysed 520 patients who received NIMV. No statistically significant differences were found in terms of age, sex, severity or gas exchange parameters amongst those patients who presented nosocomial pneumonia and complication of NIMV and those who did not [49].

**Fig. 1:**
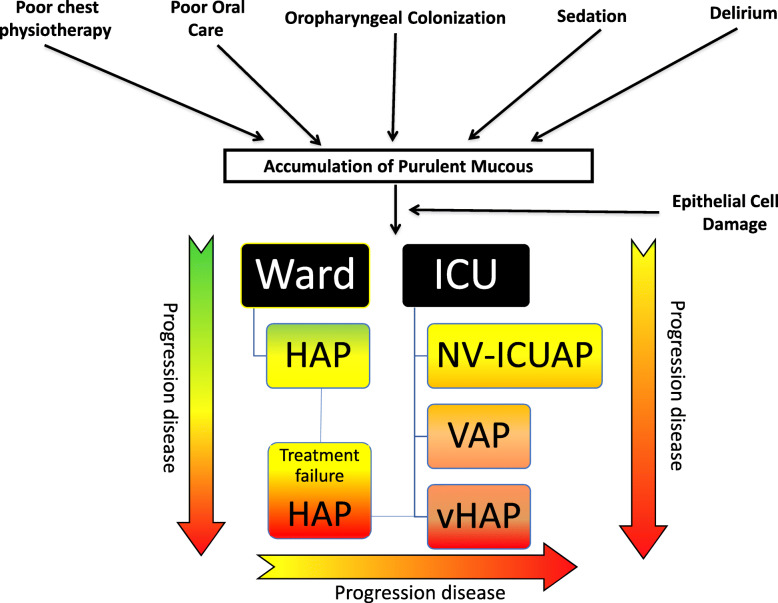
Physiopathological approach of progression of nosocomial pneumonia from wards to ICU. From green to red colour, the progression of the severity of nosocomial pneumonia is described independently of the area of hospital admission. vHAP shows the poorest outcome. HAP, hospital-acquired pneumonia; NV-ICUAP, non-ventilated acquired pneumonia; VAP, ventilator-acquired pneumonia; vHAP, ventilated hospital-acquired pneumonia

A physiopathological approach for nosocomial pneumonia has been proposed in Fig. 1.

## Prognostic factors

Pneumonia acquired in the ICU leads to a negative impact in terms of morbidity, prolonged stay and duration of MV in case of VAP and a consequent increase in healthcare cost [24]. More controversial is the direct relationship between the development of nosocomial pneumonia and increase in mortality [50, 51].

Various factors have been associated with a worse prognosis of pneumonia including the existence of comorbidities, the patient’s performance status, the infection severity at the time of its development and the patient’s response to infection. However, the study of these factors is routinely eclipsed when the same analysis is performed whether or not a suitable empirical antibiotic is used [52].

The choice of an inappropriate antibiotic treatment, which is directly related to the existence of MDROs, is probably the most relevant and, even more important, potentially modifiable prognostic factor. In fact, the likelihood of death in case of inappropriate treatment substantially increases mortality in patients with severe infections [53, 54].

Therefore, to correctly evaluate the remaining prognostic factors, it is necessary to focus the analysis on those patients who receive a suitable empirical treatment. As a second step, we must choose between two possible clinical scenarios; to consider which factors, patient and disease-related are associated with a worse final outcome or to perform a more dynamic analysis and to try to elucidate which clinical course is associated with a poor response to the treatment and, consequently, a worse final outcome. Following the first option, older age, existence of a malignant haematology disease or clinical onset in the form of septic shock or severe acute respiratory failure will be associated with higher mortality, but there is not much clinical application of this association [55]. In the same way, it occurs with analytical aspects such as initial lymphopaenia [56].

There is more interest in the evaluation of the response to early treatment strategies. Against this backdrop, Esperatti et al. validated a few years ago the association between a series of clinical variables 72 to 96 h from the onset of treatment with the prognosis of 335 patients with nosocomial pneumonia [57]. The absence of improved oxygenation, the need for mechanical ventilation in case of HAP, the persistence of fever or hypothermia together with purulent respiratory secretions, radiological worsening in more than 50% of the lung area or the development of septic shock or multi-organ failure after the onset of antibiotic treatment were more common in patients with a worse clinical course (in terms of ICU and hospital length of stay, duration of mechanical ventilation and mortality). Amongst all of these aforementioned factors, the absence of improved oxygenation was significantly associated with greater mortality (OR 2.18 [1.24–3.84] *p* = 0.007). In regard to both the original figure and course at 72–96 h of scales such as the CPIS or biomarkers such as C-reactive protein or procalcitonin, most studies agree over its prognostic use and follow-up of infection [58].

## MDROs: the link with colonization

MDR *Pseudomonas aeruginosa*, extended spectrum beta-lactamase-producing enterobacteria (ESBL-E), meticillin-resistant *Staphylococcus aureus* (MRSA), *Acinetobacter baumannii* and carbapenemase-producing *Enterobacteriaceae* (CPE) are the MDROs most commonly involved in HAP. Knowledge of local epidemiology is essential because there are significant differences in the local prevalence of each MDRO [59].

The ENVIN-HELICS report does quantify the resistance of the most important microorganisms to different antibiotics, which enables an overall vision of expected resistance rates in the case of nosocomial pneumonia in Spanish ICU [3].

The ENVIN-HELICS data also reveal an increased resistance of *Klebsiella* to carbapenems. The grade of resistance to antibiotics in the remaining bacteria has remained stable in the last few years. Table 1 shows the most important microorganisms that cause VAP and the percentage resistance to some of the main antibiotics used for these infections.

When evaluating the risk of development of nosocomial pneumonia in the ICU by a MDRO, we must first evaluate the risk factors for these pathogens. The European guidelines for nosocomial pneumonia [2] include risk factors for MDRO: septic shock, hospital ecology with high levels of MDROs, prior use of antibiotics, recent hospitalization (> 5 days) and prior colonization by MDROs. Risk factors are in general common to all MDRO; to discriminate different MDROs, we mainly base ourselves on local epidemiology and prior colonization of the patient [60]. The importance of colonization as a risk factor for suffering pneumonia by the colonizing microorganism varies according to the type of MDRO and location of the colonization. Table 2 describes the principal variables associated with resistance for the main MDROs causing NP.

**Table 2:** Principal variables associated with resistance for main MDROs causing NP

MDRO	Risk factors	References
MRSA	⇒ Age ⇒ NP appearance > 6 days after admittance ⇒ NP development excluding summers ⇒ Respiratory diseases ⇒ Multilobar involvement ⇒ Respiratory infection/colonization caused by MRSA in the previous year ⇒ Hospitalization in the previous 90 days ⇒ Recent nursing home or hospital stay ⇒ Recent exposure to fluoroquinolone or antibiotics treating Gram-positive organisms	[61–63]
*Pseudomonas aeruginosa*	⇒ Prior airway colonization by *P. aeruginosa* ⇒ Previous antibiotic treatment ⇒ Solid cancer ⇒ Shock ⇒ Alcohol abuse ⇒ Pleural effusion ⇒ Chronic liver disease independently predicted MDR amongst Pa-ICUAP ⇒ Prior use of carbapenems ⇒ Prior use of fluoroquinolones ⇒ Duration of therapy ⇒ APACHE II score	[64, 65]
KPC	⇒ Admission to ICU, antimicrobial use ⇒ Prior carbapenem ⇒ Invasive operation ⇒ Previous non-KPC-Kp infections ⇒ Duration of previous antibiotic therapy before KPC colonization	[66–69]
*Enterobacteriaceae*	⇒ Male sex ⇒ Admission from another health care facility ⇒ Ventilation at any point before culture during the index hospitalization ⇒ Receipt of any carbapenem in the prior 30 days ⇒ Receipt of any anti-MRSA agent in the prior 30 days	[68, 70]
*Acinetobacter baumannii*	⇒ APACHE II score at admission ⇒ Systemic illnesses (chronic respiratory disease and cerebrovascular accident) ⇒ Presence of excess non-invasive or invasive devices (mechanical ventilation) ⇒ Ever used antibiotics within 28 days (carbapenem and cefepime)	[5, 71, 72]

*KPC Klebsiella pneumoniae* carbapenemase, *MRSA* meticillin-resistant *Staphylococcus aureus*, *MDRO* multidrug-resistant organism, *NP* nosocomial pneumonia

## Current and future solutions

In the event of sepsis in a critically ill patient, there is an urgent need to commence an empirical antibiotic treatment that is suitable, appropriate and early [1, 2] with the risk of resistance to multiple antibiotics, which hinders complying with the premises mentioned.

The future use of rapid diagnostics is promising and will undoubtedly change our approaches to diagnosis and treatment of NP optimizing empiric antibiotic treatment. New tests have been developed such as multiplex polymerase chain reaction (MPCR), exhalome analysis and chromogenic tests [73].

MPCR has reported a sensitivity of 89.2% and a specificity of 97.1%, using BAL samples, and 71.8% sensitivity and 96.6% (range, 95.4–97.5%) using endotracheal aspirates (ETA) [74].

In the MAGIC-BULLET study, Filmarray® showed a sensitivity of 78.6%, an specificity of 98.1%, a positive predictive value of 78.6% and a negative predictive value of 96.6% in respiratory samples. Furthermore, Filmarray® provided results within only 1 h directly from respiratory samples with minimal sample processing times [34].

A new score (CarbaSCORE) was recently published; its aim is to identify those critically ill patients who will need to be treated with a carbapenem with the intention of using these antibiotics more selectively [75]. This consideration is appropriate, however, ascertaining some of the variables necessary, such as the existence of bacteraemia or colonization by MDROs involves a delay, which cannot be assumed in the septic patient.

**Fig. 2:**
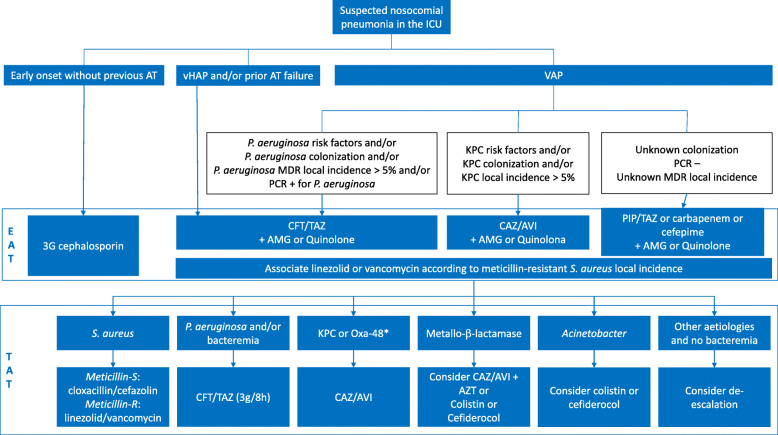
PANNUCI algorithm. From empirical to targeted treatment on nosocomial pneumonia in ICU. After analyzing the onset, the previous use of antimicrobials or clinical condition (vHAP or VAP), empirical antimicrobial therapy is chosen based on risk factors, previous colonization, local flora and/or use of rapid techniques. Therefore, targeted therapy is selected depending on the type of microorganism isolated and the possible advantages of one antimicrobial over others. AT, antimicrobial therapy; vHAP, ventilated hospital-acquired pneumonia; VAP, ventilator-associated pneumonia; MDR, multidrug-resistant; PCR, polymerase chain reaction; CFT/TAZ, ceftolozane/tazobactam; CAZ/AVI, ceftazidime/avibactam; PIP/TAZ, piperacillin/tazobactam; AMG, aminoglycoside; AZT, aztreonam; EAT, empirical antimicrobial treatment; TAT, targeted antimicrobial treatment; OXA-48, OXA-48 carbapenemase; KPC, *Klebsiella pneumoniae* carbapenemase; R, resistance. *If Oxa-48 susceptible to CAZ/AVI

An algorithm that includes the priorities analysed to update empirical and targeted treatment in critically ill patients has been designed (Fig. 2) after reviewing the major randomized, controlled clinical trials of antimicrobial agents actually available for NP in the last 10 years [76–84] (Table 3) and the considerations made before about epidemiology (Table 1), antimicrobial resistances (Table 2), rapid microbiological test and risk factors for HAP.

**Table 3:** List of major randomized, controlled clinical trials of systemic antimicrobial agents actually available for treating NP in the last 10 years

Author, year, name of the trial	Antimicrobial tested and comparator	Phase, blinded, design	Microorganism	Subject	Primary outcome	Results of primary outcome	Mortality	Comments
Freire, 2010 [76]	Tigecycline (T) Imipenem (I)	III, yes, NI	All pathogens	HAP + VAP	Clinical response in CE and c-mITT populations at TOC	c-mITT: T, 62.7%; I, 67.6% CE: T, 67.9%; I, 78.2%	T, 14.1% I, 12.2%	T was non-inferior to I for c-mITT but not the CE population due to the results in VAP. FDA warning against T use for VAP.
Rubinstein, 2011, ATTAIN 1 and 2 [60]	Telavancin (Te) Vancomycin (V)	III, yes, NI	Gram-positive	HAP	Clinical response at FU/TOC	AT: Te, 58.9%; V, 59.5% CE: Te, 82.4%; V, 80.7%	Te, 21.5% V, 16.6%	Increases in serum creatinine level were more common in the telavancin group.
Kollef, 2012 [78]	Doripenem (D), 7 days Imipenem (I), 10 days	IV, yes, NI	All pathogens	VAP	Clinical cure at EOT (day 10) in the MITT	D, 45.6% I, 56.8%	D, 21.5% I, 14.8%	Non-inferiority of a fixed 7-day treatment with D was no achieved FDA warning against D use for VAP.
Wunderink, 2012, ZEPHIR [79]	Linezolid (L) Vancomycin (V)	IV, yes, NI	Meticillin-resistant *Staphylococcus aureus*	HAP + VAP	Clinical outcome at EOS in PP patients	L, 57.6% V, 46.5%	L, 15.7% V, 17%	Nephrotoxicity occurred more frequently with V.
Ramirez, 2013 [80]	Tigecycline low dose (TLD) Tigecycline high dose (THD) Imipenem	II, yes, NI	All pathogens	HAP + VAP	Clinical response at EOT	THD, 85% TLD, 69.6 I, 75%	–	THD could be necessary to treat HAP/VAP.
Awad, 2014 [81]	Ceftobiprole medocaril (C) Ceftazidime + Linezolid (CAZ/L)	III, yes, NI	All pathogens	HAP + VAP	Clinical cure at the TOC	ITT: C, 49.9%; CAZ/L, 52.8% CE: C, 69.3%; CAZ/L, 71.3%	C, 16.7% CAZ/L, 18%	Non-inferiority of C compared with CAZ/L was not demonstrated in VAP patients.
Torres, 2018, REPROVE [82]	Ceftazidime/avibactam (CAZ/AVI) Meropenem (M)	III, yes, NI	All pathogens	HAP + VAP	Clinical cure at the TOC	c-mITT: CAZ/AVI, 68.8%; M, 73% CE: CAZ/AVI, 77.4%; M, 78.1%	CAZ/AVI, 8.1% M, 6.8%	CAZ/AVI could be a potential alternative to carbapenems in HAP/VAP patients.
Kollef 2019, ASPECT-NP [83]	Ceftolozane/tazobactam (CFT-TAZ) Meropenem	III, yes, NI	All pathogens	HAP + VAP, only patients on MV	28-day all-cause mortality in ITT	CFT-TAZ, 24% M, 25.3%	CFT-TAZ, 24% M, 25.3%	In HAP and in those in whom previous antibacterial therapy was unsuccessful, CFT-TAZ showed lower mortality.
Cisneros, 2019, Magic-Bullet [84]	Colistin (Co) Meropenen (M)	IV, no, NI	All pathogens	Late VAP	Mortality at 28 days after randomization in mMITT	Co, 23.2% M, 25.3%	Co, 23.2% M, 25.3%	The study was interrupted after the interim analysis due to excessive nephrotoxicity in the colistin group (33:3% vs 18.8%).

*AT* all treated patients, *CAZ/AVI* ceftazidime/avibactam, *CE* clinically evaluable population, *CFT-TAZ* ceftolozane/tazobactam, *Co* colistin, *c-mITT* clinical modified intent-to-treat population, *D* doripenem, *EOS* end of study, *EOT* end of treatment, *FU* follow-up, *I* imipenem, *ITT* intention-to-treat population, *M* meropenem, *MITT* modified intent-to-treat population, *mMITT* microbiologically modified intention-to-treat population, *MV* mechanical ventilation, *NI* non-inferiority, *T* tigecycline, *Te* telavancin, *TOC* test of cure, *THD* tigecycline high dose, *TLD* tigecycline low dose, *PP* evaluable per-protocol, *V* vancomycin

Some new antibiotics have been recommended over old ones based on their potential advantages shown in pivotal studies (Table 3), observational studies and in vitro data. However, the use of other families of antibiotics has been also warranted.

Various experts recommend using these new antibiotics according to the site of infection, clinical severity, existence of risk factors for MDRO acquisition, existence of comorbidities and existing MDROs in each unit/hospital as suggested in the algorithm [4, 5, 85–87].

The onset of two antibiotics such as ceftolozane/tazobactam (CFT-TAZ) and ceftazidime/avibactam (CAZ/AVI) has broadened the treatment options for patients with suspected MDRO infection. Both antibiotics offer some advantages: apart from the demonstrated efficacy in clinical trials for approval, they present a better in vitro activity and less resistance and can also be used within the scope of an antibiotic policy aimed to reserve carbapenems [4, 5].

Because of its specific features, all authors included in this point of view manuscript coincided in the choice of CFT/TAZ to treat *P. aeruginosa* [85, 86] infections and CAZ/AVI for infections caused by KPC-like carbapenemase-producing *Enterobacteriaceae* [87]. However, they acknowledged that both antibiotics have never been compared head to head.

CFT/TAZ presents greater in vitro activity against *P. aeruginosa*, with less resistance than the remaining current anti-pseudomonal agents in global terms [88]. CFT/TAZ also exhibits the lowest mutant prevention concentration (MPC) against *P. aeruginosa*, as well as colistin and quinolones (2 mg/L) [85]. The clinical trial ASPECT-NP [83] reveals a favourable result for patients who suffer from HAP that require invasive MV treated with CFT/TAZ (mortality at 28 days, 24.2% vs 37%) and also in those patients in whom initial antibiotic treatment failed (mortality at 28 days, 22.6% vs 45%). In patients with bacteraemia, a trend towards a higher rate of clinical cure (10.5% vs 36%), without statistical significance, was observed in CFT/TAZ-treated patients. In this clinical trial, higher levels of microbiological cure in pneumonia caused by *P. aeruginosa* were also observed in patients who received CFT/TAZ.

On the other hand, CAZ/AVI was associated with better survival rates in patients with bacteraemia who required rescue treatment in infections caused by KPC-producing *Enterobacteriaceae* [89]. In case of infection caused by a CAZ/AVI-susceptible OXA-48 strain, CAZ/AVI could be an option to treat it [90]. Data extracted from an in vitro study suggest that CAZ/AVI plus aztreonam could be an option to treat infections caused by metallo-β-lactamase-producing *Enterobacteriaceae* [91].

The MERINO Trial [92] randomized patients hospitalized with bacteraemia caused by enterobacteria resistant to ceftriaxone to receive antibiotic treatment with meropenem or piperacillin/tazobactam. The clinical outcomes were unfavourable for the group of patients that received piperacillin/tazobactam, which cuts down the treatment options for these infections. In published clinical trials, both CFT/TAZ and CAZ/AVI [82, 83] antibiotics demonstrated appropriate activity and clinical efficacy to ESBL-E, whereby they arise as a new alternative and may be included in carbapenem-spare regimens.

Cefiderocol recently received US Food and Drug Administration’s (FDA) approval for the treatment of complicated urinary tract infections, including pyelonephritis, and is currently being evaluated in phase III trials for treating nosocomial pneumonia and infections caused by carbapenem-resistant Gram-negative pathogens including *Acinetobacter* spp. [93].

Colistin is really a non-effective drug to consider for HAP unless aerosolized. The Magic Bullet trial failed to demonstrate non-inferiority of colistin compared with meropenem, both combined with levofloxacin, in terms of efficacy in the empirical treatment of late VAP but showed the greater nephrotoxicity of colistin [84]. However, sometimes, especially in VAP caused by MDR *Acinetobacter baumannii*, no other options are available. Other antimicrobials such as ceftobiprole or tigecycline have not been considered due to the failure to demonstrate non-inferiority in some of the trials reviewed (Table 3).

The use of aerosolized therapy for VAP is still controversial. Two recent multicenter, randomized, double-blinded, placebo-controlled trials of adjunctive nebulized antibiotics for VAP patients with suspected MDR Gram-negative pneumonia were negative to achieve their primary endpoints [94, 95]. For this reason, their use as an adjunctive therapy cannot be supported. Rescue therapy for MDROs might be considered when systemic therapy failed [96].

Antibiotic stewardship and duration of antibiotic therapy also deserve our attention. The clinical severity of a suspected VAP makes intensivists start as soon as possible broad-spectrum antimicrobial therapy when, in fact, many patients treated do not have NP. Clinical scores, such as Clinical Pulmonary Infection Score (CPIS), or non-specific biomarkers such procalcitonin (PCT) and C-reactive protein (CRP) must be applied to begin or to stop antibiotic treatment as previously discussed [73].

Prolonged courses of antimicrobial therapy promote more resistance. European guidelines recommend antibiotic treatment for HAP no longer than 7 days [2]. However, the duration of therapy for MDROs is not clearly established. A new trial (iDIAPASON) is trying to demonstrate that a shorter therapy strategy in *Pseudomonas aeruginosa-*VAP treatment is safe and not associated with an increased mortality or recurrence rate [97]. This strategy could lead to decreased antibiotic exposure during hospitalization in the ICU and in turn reduce the acquisition and the spread of MDROs.

## Conclusions

Determining the risk factor for nosocomial pneumonia is one of the pillars for the antibiotic selection. There are different risk factors: patient-related (prolonged hospital length of stay and comorbidities, use of prior antibiotics and septic shock), procedure-related (deficient hand hygiene or inappropriate care of respiratory support devices) and intervention-related (immunosuppressants and prolonged/inappropriate antibiotic treatment). Antibiotic treatment (including new ones) must be administered early and be appropriate. These aspects are key to VAP outcomes because of the severity of patients and the possible onset of MDROs.

## Data Availability

Not applicable.

## Abbreviations

3°G cef: 3° generation cephalosporin
AT: Antimicrobial therapy
AMG: Aminoglycoside
ARDS: Acute respiratory distress syndrome
ASP: Antimicrobial stewardship programmes
AZT: Aztreonam
CPE: Carbapenemase-producing *Enterobacteriaceae*
CAZ/AVI: Ceftazidime/avibactam
CFT/TAZ: Ceftolozane/tazobactam
COPD: Chronic obstructive pulmonary disease
CRP: C-reactive protein
EAT: Empirical antimicrobial treatment
ECDC: European Centre for Disease Prevention and Control
ENVIN-HELICS: National Surveillance Programme of Intensive Care Unit (ICU)-Acquired Infection in Europe Link for Infection Control through Surveillance
ESBL-E: Extended spectrum beta-lactamase-producing enterobacteria
FDA: Food and Drug Administration
HAP: Hospital-acquired pneumonia
KPC: *Klebsiella pneumoniae* carbapenemase
MDR: Multidrug resistant
MDROs: Multidrug-resistant organisms
MRSA: Meticillin-resistant *Staphylococcus aureus*
MV: Mechanical ventilation
NIMV: Non-invasive mechanical ventilation
NV-ICUAP: Non-ventilated acquired pneumonia
OXA-48: OXA-48 carbapenemase
PCR: Polymerase chain reaction
PCT: Procalcitonin
PIP/TAZ: Piperacillin/tazobactam
POCT: Point of care test
RCT: Randomized clinical trials
VA-ECMO: Veno-arterial extracorporeal membrane oxygenation
VAP: Ventilator-acquired pneumonia
VAT: Ventilator-associated tracheobronchitis
vHAP: Ventilated hospital-acquired pneumonia
TAT: Targeted antimicrobial treatment
